# The Crosstalk Between Immune Infiltration, Circulating Tumor Cells, and Metastasis in Pancreatic Cancer: Identification of HMGB3 From a Multiple Omics Analysis

**DOI:** 10.3389/fgene.2022.892177

**Published:** 2022-06-08

**Authors:** Hao-dong Tang, Yang Wang, Peng Xie, Si-yuan Tan, Hai-feng Li, Hao Shen, Zheng Zhang, Zheng-qing Lei, Jia-hua Zhou

**Affiliations:** ^1^ Department of Surgery, School of Medicine, Southeast University, Nanjing, China; ^2^ Department of Hepato-Pancreatico-Biliary Surgery, Zhongda Hospital Southeast University, Nanjing, China

**Keywords:** circulating tumor cells (CTCs), pancreatic ductal adenocarcinoma (PDAC), CIBERSORT, weighted gene co-expression network analysis (WGCNA), single-cell RNA-sequencing (scRNA-seq)

## Abstract

Metastasis is the major cause of death in patients with pancreatic ductal adenocarcinoma (PDAC), and circulating tumor cells (CTCs) play an important role in the development of metastasis. However, few studies have uncovered the metastasis mechanism of PDAC based on CTCs. In this study, the existing bulk RNA-sequencing (bulk RNA-seq) and single-cell sequencing (scRNA-seq) data for CTCs in pancreatic cancer were obtained from the Gene Expression Omnibus (GEO) database. Analysis of tumor-infiltrating immune cells (TIICs) by CIBERSORT showed that the CTCs enriched from the peripheral blood of metastatic PDAC were found to contain a high proportion of T cell regulators (Tregs) and macrophages, while the proportion of dendritic cells (DCs) was lower than that enriched from localized PDAC. Through weighted gene co-expression network analysis (WGCNA) and the result of scRNA-seq, we identified the hub module (265 genes) and 87 marker genes, respectively, which were highly associated with metastasis. The results of functional enrichment analysis indicated that the two gene sets mentioned above are mainly involved in cell adhesion and cytoskeleton and epithelial–mesenchymal transition (EMT). Finally, we found that HMGB3 was the hub gene according to the Venn diagram. The expression of HMGB3 in PDAC was significantly higher than that in normal tissues (protein and mRNA levels). HMGB3 expression was significantly positively correlated with both EMT-related molecules and CTC cluster–related markers. Furthermore, it was also found that HMGB3 mutations were favorably related to tumor-associated immune cells through the TIMER2.0 online tool. We further demonstrated that PDAC patients with higher HMGB3 expression had significantly worse overall survival (OS) in multiple datasets. In summary, our study suggests that HMGB3 is a hub gene associated with EMT in CTCs, the formation of CTC clusters, and infiltration patterns of immune cells favorable for tumor progression and metastasis to distant organs.

## Introduction

Pancreatic cancer is an aggressive and fatal disease. According to the American Cancer Society (ACS), there are approximately 60,430 new cases of pancreatic cancer and approximately 48,220 deaths in the United States in 2021 (Siegel et al., 2021). Pancreatic cancer could become the second leading cause of cancer death by 2030 (Rahib et al., 2014). Pancreatic ductal adenocarcinoma (PDAC) is the most common pathological type, accounting for more than 90% of pancreatic cancer cases (Hidalgo and Von Hoff, 2012). At present, surgical resection is still the only method to cure PDAC. However, due to its insidious onset and lack of biological markers for early diagnosis, most patients already acquire distant metastasis by the time the diagnosis is confirmed, resulting in less than 9% of the 5-year survival rate (Chen et al., 2016; Siegel et al., 2020). Although localized PDAC patients undergo radical resection, many patients still suffer from early recurrence or even metastasis, contributing to more than 90% of cancer deaths worldwide. Metastasis of tumors has been widely concerning. In 1869, John Ashworth discovered that there were tumor cells in the blood of patients with metastatic cancer histologically similar to the primary tumor and proposed the concept of circulating tumor cell (CTC) (Ashworth, 1869). CTC is a complete tumor cell, which carries multiple omics information (genome, transcriptome, proteome, metabolome, etc.) and is considered a precursor cell of metastasis. It is detached from the primary tumor and enters peripheral blood circulation to form distant lesions (Keller and Pantel, 2019). A plethora of studies have shown that increased CTC number indicates malignant clinicopathological features of the tumor (Liao et al., 2014; Wang et al., 2017; Miyamoto et al., 2018). The detection rate of CTC was higher in patients with metastatic tumor than in those with primary tumor (Goodman et al., 2018). The number of CTCs could be used to evaluate the therapeutic effect and prognosis for various kinds of cancer (Goldkorn et al., 2014; Huang et al., 2015; Cui et al., 2020). A meta-analysis conducted by our research center also found the value of CTC in predicting the prognosis of patients with pancreatic cancer (Wang et al., 2020). However, with the development of RNA-sequencing technology, it is gradually found that it is insufficient to only focus on the number of CTCs. An increasing number of studies have found that CTC has various specific phenotypes (circulating cancer stem cells, CTC with epithelial–mesenchymal transition and CTC clusters, etc.,), which might be the key factor affecting tumor metastasis and prognosis (Barriere et al., 2014; Fabisiewicz and Grzybowska, 2017; Agnoletto et al., 2019).

In addition, the tumor microenvironment has been increasingly significant in immunotherapy for cancer in recent years, and multiple studies have found that the proportion and functional changes of tumor-infiltrating immune cells (TIICs) are related to the initiation, progression, and prognosis of PDAC (Chen et al., 2020; Chen et al., 2021). CTCs are released from the primary site and are more vulnerable to attack by immune cells as they circulate in the peripheral blood. However, previous research has revealed that CTCs induce a local microenvironment, which is conducive to CTCs evading immune surveillance to survive (Fabisiewicz and Grzybowska, 2017). Therefore, CTCs that could eventually survive and lead to metastasis might have a special microenvironment of immune infiltration and gene expression profile.

Unfortunately, few studies have been conducted to study immune cell infiltration, tumor metastasis, and proliferation based on CTCs. The aim of the present study is to investigate patterns of infiltrating immune cells and key gene and pathways associated with metastasis in pancreatic CTCs using CIBERSORT analysis, weighted gene co-expression network analysis (WGCNA), principal component analysis (PCA), and T-stochastic neighbor embedding (TSNE) analysis. Taking it as a new target to interfere with the metastasis of cancer could provide a new solution for cancer treatment.

## Materials and Methods

### Data Sources

The gene sequencing data related to PDAC metastasis were retrieved from the GEO database (https://www.ncbi.nlm.nih.gov/geo/). Finally, GSE144561 (Franses et al., 2020) and GSE114704 (Dimitrov-Markov et al., 2020) were selected for subsequent analysis. GSE144561 included RNA-sequencing datasets of healthy donors (21 normal samples) and PDAC patients (42 localized PDAC samples and 18 metastatic PDAC samples) of CTC samples processed by using microfluidic CTC-iChip. All samples were generated on the Illumina NextSeq 500 (Homo sapiens) on the GPL18573 platform.

Another scRNA-seq dataset GSE114704 included 10 CTCs, 23 liver metastasis (LM) cells, and 37 primary tumor (PT) cells derived from metastatic PDAC patient–derived xenograft (PDX) mouse models. The enrichment and separation of CTCs were also conducted by using microfluidic CTC-iChip. All samples were based on the Illumina HiSeq 2500 (Homo sapiens) on the GPL16791 platform. Two sets of gene sequencing data can be accessed for free, and detailed information on both datasets is available in the Supplementary File.

### Analysis of Infiltrating Immune Cell Patterns

CIBERSORT is an analytical tool developed by Newman et al. to quantify infiltrating immune cell portions based on normalized RNA gene expression profiles (Newman et al., 2015). We used “CIBERSORT” (R package) to analyze the 22 immune cell compositions of PDAC patients in the dataset GSE14456 (42 local PDAC and 18 metastatic PDAC). To improve the accuracy of the algorithm, we set 1000 aligned default signature matrices. The relative proportions of 22 infiltrating immune cells and CIBERSORT *p*-value were evaluated for each sample. Furthermore, samples with a CIBERSORT *p* < 0.05 were selected for subsequent analysis.

### Acquisition of Differentially Expressed Genes

The genes with mean read counts over 1 in all samples were included in the following analysis. Then, DEGs were screened from read counts of 21,253 genes between healthy donors and local PDAC patients (GSE14456) using the “DESeq2” R package (Love et al., 2014), with adjusted *p* < 0.05 & |log2FC|>2. The read counts of 23,049 genes between healthy donors and metastatic PDAC patients were also processed according to the same criteria. Finally, two DEG sets were merged to represent the DEGs between healthy donors and PDAC patients.

### Weighted Gene Co-Expression Network Analysis and Identification of Modules Associated With Metastasis

We used the R package “WGCNA” to build the coexpression network of genes in localized PDAC and metastatic PDAC patients (Langfelder and Horvath, 2008). First, a scale-free network was constructed. Then, the function pick-soft threshold was used to select the appropriate soft threshold β. Subsequently, the soft threshold β was applied for the transformation of Pearson’s correlation matrices to a weighted adjacency matrix. The adjacency matrix was transformed into a topological overlap matrix (TOM) to estimate network connectivity. In the next part, we used the dynamic tree-cut algorithm to cluster dendrogram branches into several modules and assigned them colors. The minimum module size was set at 20, with the module detection sensitivity deepSplit 2, and the modules with larger than 0.85 pairwise correlation were merged. Finally, Pearson’s correlations between module eigengenes (MEs) and clinical trait were calculated to identify the module that was relevant to metastasis. Only the module with the highest significant positivity of correlation with metastasis was included in the subsequent study.

### Principal Component Analysis and T-Stochastic Neighbor Embedding Analysis

Before analysis, “Seurat”, a specific R toolkit, was used for quality control (QC) of single-cell transcriptomic data (GSE114704) (Stuart et al., 2019). We used a similar pipeline for following data analysis according to the previous study (Wu et al., 2020). To remove low-quality cells, we adopted strict filtering standards for each individual (for example, min. cells = 3; min. features = 50; nFeature_RNA > 50; percent. mt < 5%). Subsequently, the remaining transcriptomic data of each individual were log-normalized with a multiplied scale factor of 10,000 to perform a global-scaling normalization, and the “vst” method was then used to select the top 5,000 highly variable genes based on mean variance for downstream PCA analysis. Twenty principal components (PCs) were obtained by PCA analysis. Then, cluster analysis was performed on the cells in these 20 PCs to determine which PCs have the most significant difference. Then, these PCs with significant difference were subjected to TSNE analysis to define the different clusters. The genes among distinct clusters were then differentially analyzed for identification of marker genes (adjusted *p* < 0.05 and |log2FC|>0.5).

### Functional Enrichment Analysis

The Metascape tool (https://metascape.org) (Zhou et al., 2019) was used to perform Gene Ontology (GO) biological processes (BP), cell components (CC), molecular functions (MF), Hallmark Gene Sets, and Kyoto Encyclopedia of Genes and Genomes (KEGG) pathway enrichment analysis of module genes obtained by WGCNA analysis and marker genes identified by distinct clusters. In screening for enrichment information, *p* < 0.05 was considered statistically significant.

### Definition of the Hub Gene

The genes coexpressed in module genes and marker genes were filtrated as hub genes correlated with metastasis through the Wayne diagram tool (http://bioinformatics.psb.ugent.be/webtools/Venn/). To confirm the importance of hub genes, we evaluated their expression levels in pancreatic cancer and normal tissues by The Gene Expression Profiling Interactive Analysis 2 (GEPIA2) (http://gepia.cancer-pku.cn/index.html). The protein expression levels of the hub gene in the pancreatic tumor and nontumor tissues were determined using the Human Protein Atlas (HPA) (https://www.proteinatlas.org/) (Asplund et al., 2012). We investigated whether the expression levels of hub genes correlated with clinical prognosis (overall survival, OS) in the pancreatic adenocarcinoma (PAAD) cohort by long-term outcome and gene expression profiling database of pan-cancers (LOGpc) (http://bioinfo.henu.edu.cn/DatabaseList.jsp) (Zhang etal., 2020). Furthermore, we analyzed the correlation between the key gene and EMT-related molecules and CTC cluster–related markers in the GEO and GEPIA2 databases, respectively. The correlation analysis between the key gene and immune cells was carried out through the TIMER2.0 database, which is a comprehensive and freely accessible data source (Li et al., 2020).

### Statistical Analysis

Correlations of gene expression were established using Spearman’s correlation coefficient. Associations between categorical variables were tested using the Wilcoxon test. All statistical analyses were performed using R software (version 4.1.0). When *p* < 0.05, the difference was considered statistically significant.

## Results

### Immune Microenvironment of CTCs in PDAC Patients

**FIGURE 1:**
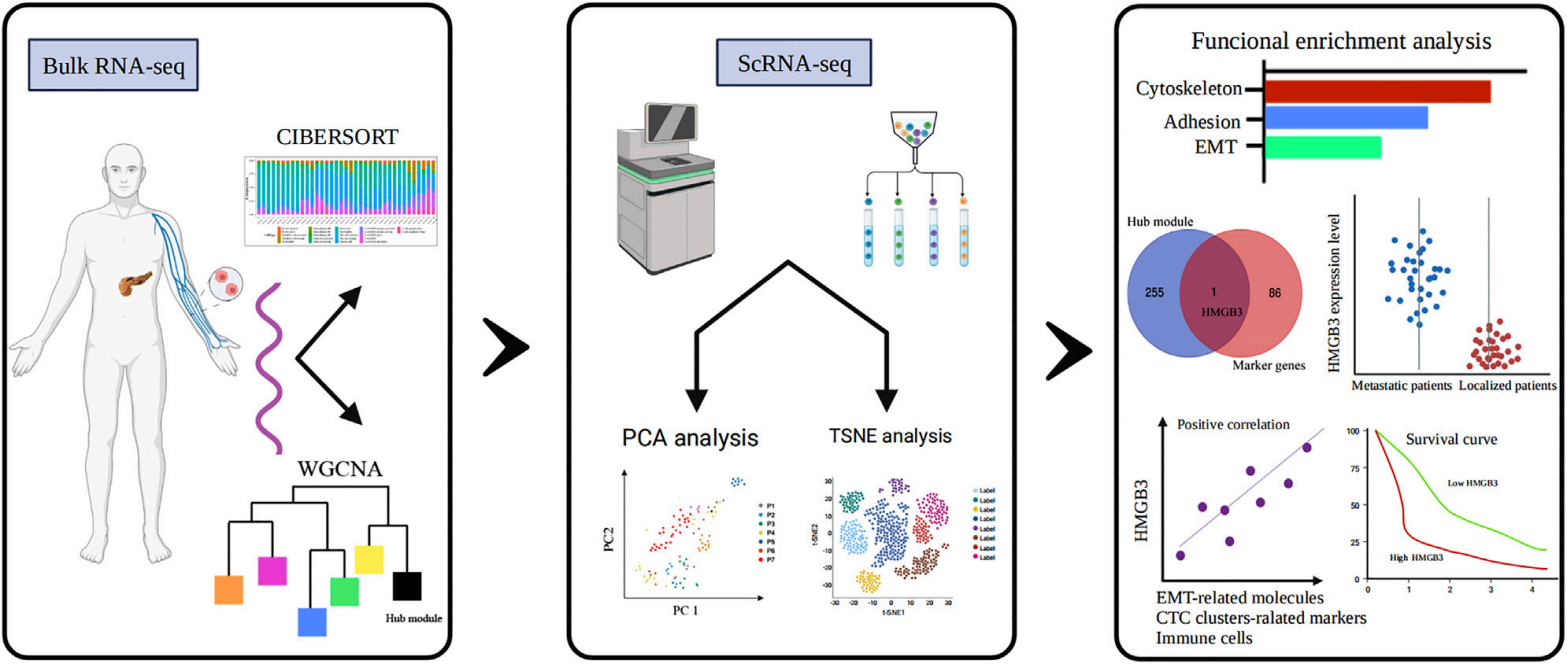
Study flow diagram. WGCNA, weighted gene co-expression network analysis; PCA, principal component analysis; TSNE, T-stochastic neighbor embedding; HMGB3, high-mobility group box 3.

**FIGURE 2:**
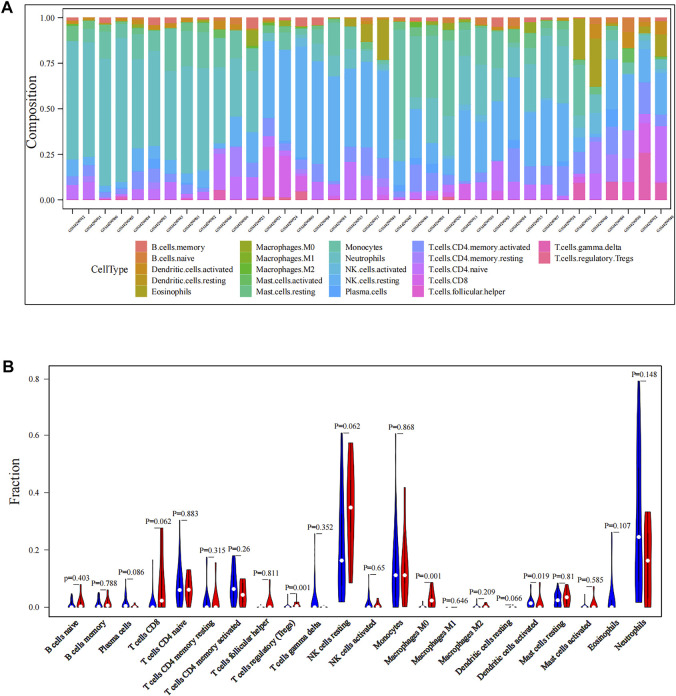
Immune infiltration levels of CTCs in PDAC patients. **(A)** Landscape of immune infiltration in CTCs of PDAC patients. **(B)** Analysis of differential immune cells between localized PDAC and metastatic PDAC. CTCs, circulating tumor cells; PDAC, pancreatic ductal adenocarcinoma.

The workflow of all analyses involved in this study is depicted in Figure 1. First, we explored proportions of the 22 TIICs in CTCs from 42 localized PDAC patients and 18 metastatic PDAC patients (GSE14456) using the CIBERSORT algorithm. Under screening criteria of CIBERSORT *p* < 0.05, only 37 samples were qualified in this study, and the landscape of immune infiltrations in CTCs is summarized in Figure 2A. Subsequently, we investigated the difference of TIICs in CTCs with localized PDAC and metastatic PDAC. Apparently, metastatic PDAC had more Tregs and M0 macrophages than localized PDAC (*p* = 0001; *p* = 0001). On the contrary, DCs were found to be significantly lower (*p* = 0.019). In addition, compared with localized PDAC, the proportions of CD8^+^ T cells and NK cells were higher in metastatic PDAC, whereas infiltrations of plasma cells were lower. Unfortunately, there were no statistically significant differences (*p* = 0.062, *p* = 0.062, and *p* = 0.086) (Figure 2B). The high proportion of Tregs and macrophages infiltrating CTCs might be responsible for protecting CTCs from immune system attack in circulating blood and ultimately leading to metastasis.

### Identification of DEGs Between Healthy Donors and PDAC Patients

**FIGURE 3:**
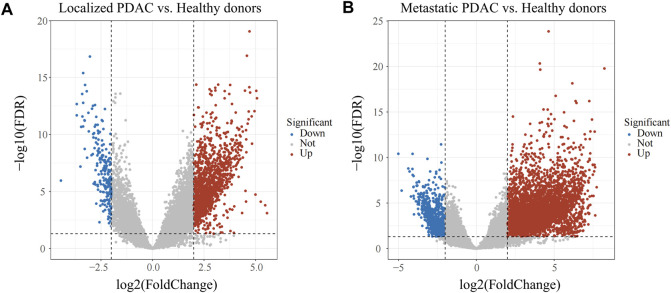
Volcano plot of DEGs in GSE14456. **(A)** DEGs between localized PDAC patients and healthy donors. **(B)** DEGs between metastatic PDAC patients and healthy donors. PDAC, pancreatic ductal adenocarcinoma; DEGs, differentially expressed genes.

The discrepancies in immune cell infiltration patterns of CTCs between localized PDAC and metastatic PDAC have been indicated, and then we further explored differences in RNA transcriptome expression between the two groups. First, with adjusted *p* < 0.05 and |log2FC|> 2 as the screening conditions, 1699 DEGs were screened in localized PDAC patients (GSE14456, N = 42) compared with healthy donors (GSE14456, N = 21) (Figure 3A). Moreover, in metastatic PDAC patients (GSE14456, N = 18), 6,135 DEGs were identified (Figure 3B). Two DGE sets that were previously mentioned were merged, and then duplicates were removed to retain the unique genes (N = 7,452) for subsequent WGCNA analysis.

### WGCNA and Identification of Modules Associated With Metastasis

**FIGURE 4:**
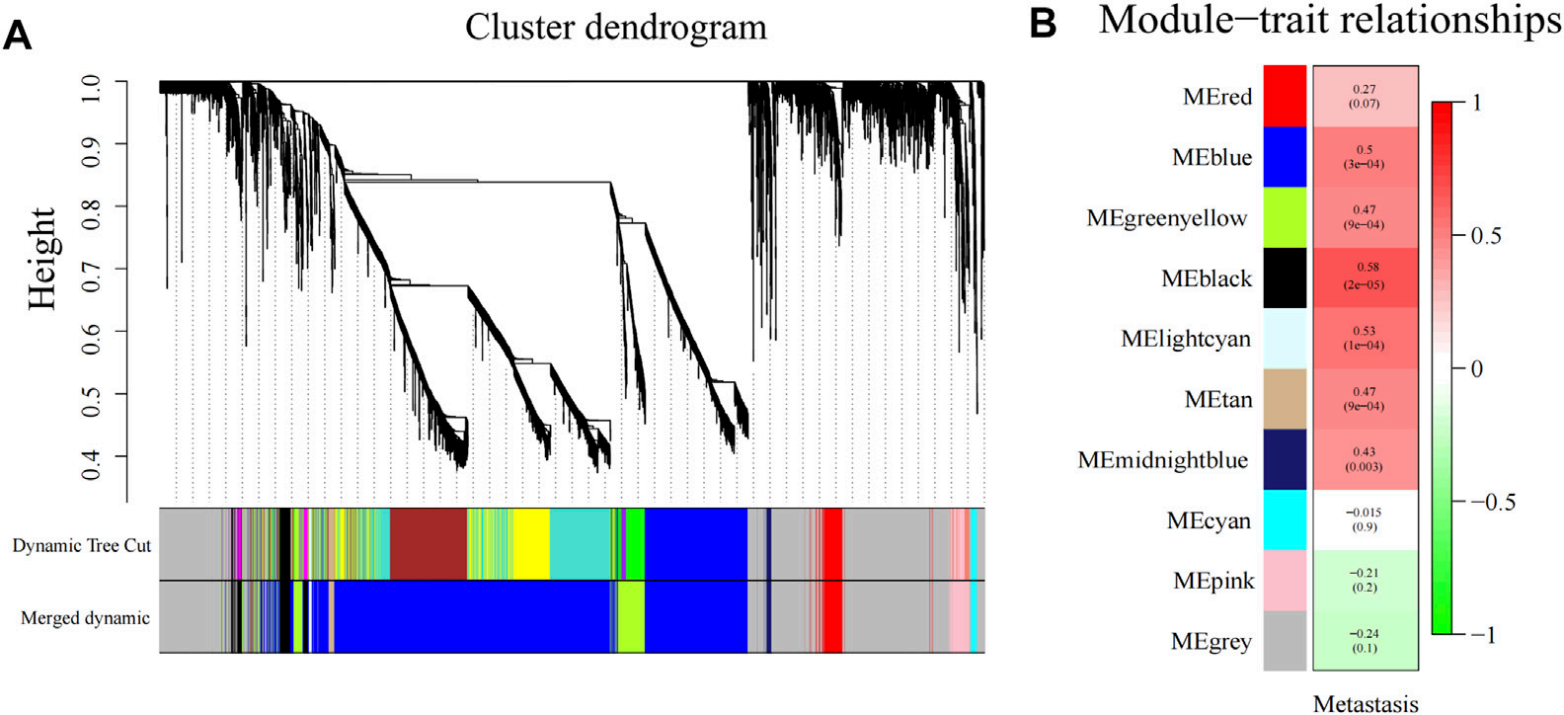
Gene modules identified by WGCNA. **(A)** Gene dendrogram obtained by clustering the dissimilarity based on consensus topological overlap with the corresponding module colors indicated by the color row. **(B)** Heatmap of correlation between the module eigengenes and gene modules. WGCNA, weighted gene co-expression network analysis; ME, module eigengene.

First, the “Hclust” function was used for sample clustering analysis of PDAC (localized PDAC 42 and metastatic PDAC 18), and 13 outliers were removed according to the threshold value (cut height = 10,000) (Supplementary Figure S1). Then, the soft threshold β was determined by the “sft$powerEstimate” function and set to 26 (Supplementary Figure S2). After TOM network construction, the modules with larger than 0.85 pairwise correlation were merged (cut height as 0.15, Supplementary Figure S3) and 10 gene co-expression modules were detected and assigned colors with sizes between 43 and 3,745 genes (Figure 4A). The gray module is the set of genes that cannot be aggregated together. Then, Pearson’s correlation between different modules and clinical trait (metastasis) was calculated, and the black module has the highest significant positive correlation with metastasis (Cor = 0.58, *p* < 0.001) (Figure 4B). There were 256 genes in the black module.

### PCA and TSNE Analysis

**FIGURE 5:**
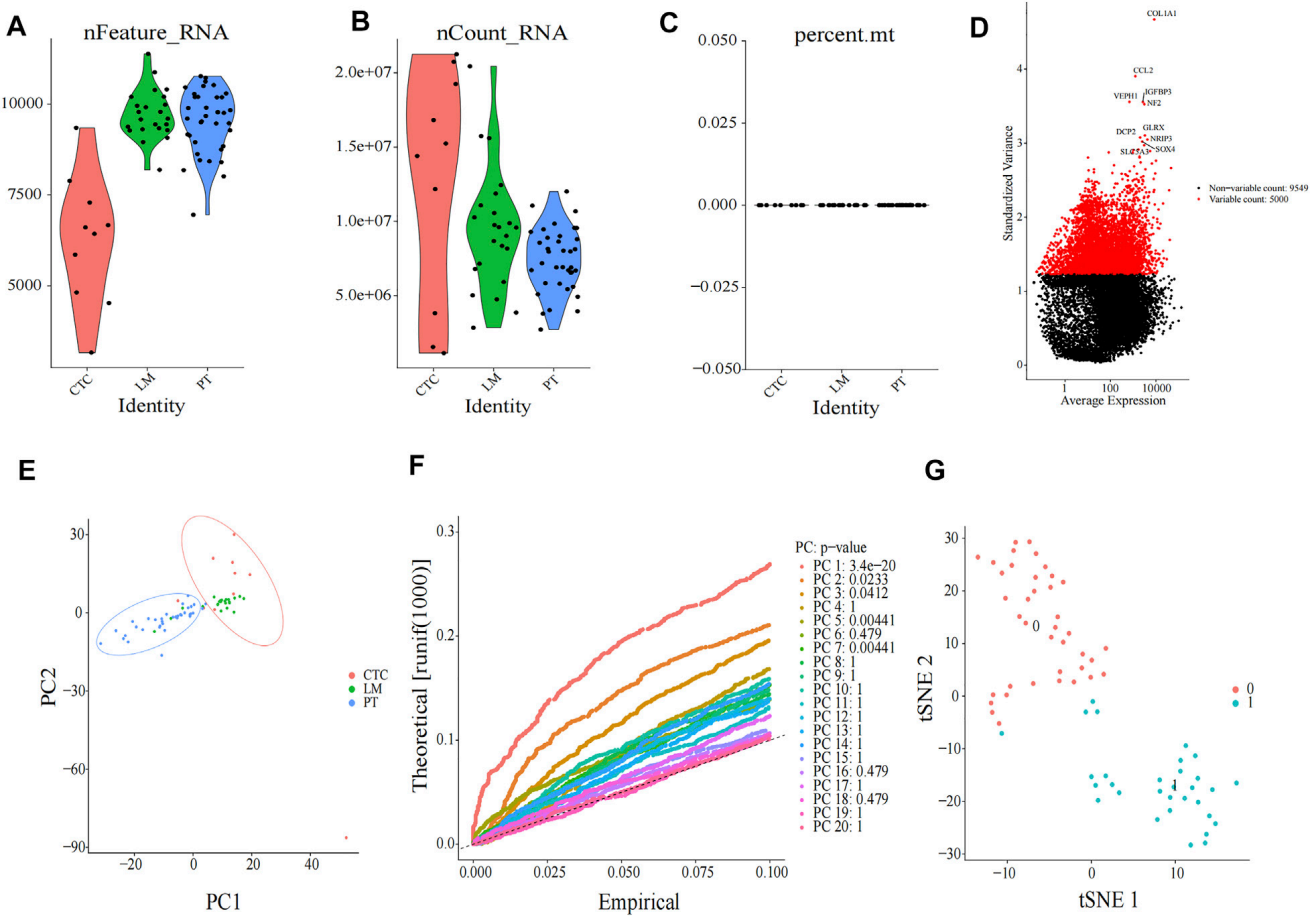
Clustering of cells after single-cell sequencing. Quality check of 70 single-cell sequencing in **(A)** nFeature, **(B)** nCount, and **(C)** percent mt three aspects, as shown in violin plots. **(D)** Scatter diagram of larger expression alteration in different cells and the top 5,000 were marked red pots. **(E)** PCA of 70 cells. **(F)** JackStraw plot of the first 20 PCs. **(G)** t-SNE plot of 70 cells. All cells can be distributed into two clusters (cluster 0 and cluster 1). CTC, circulating tumor cell; LM, liver metastasis; PT, primary tumor; PCA, principal component analysis; PC, principal components; tSNE, t-Stochastic Neighbor Embedding.

**FIGURE 6:**
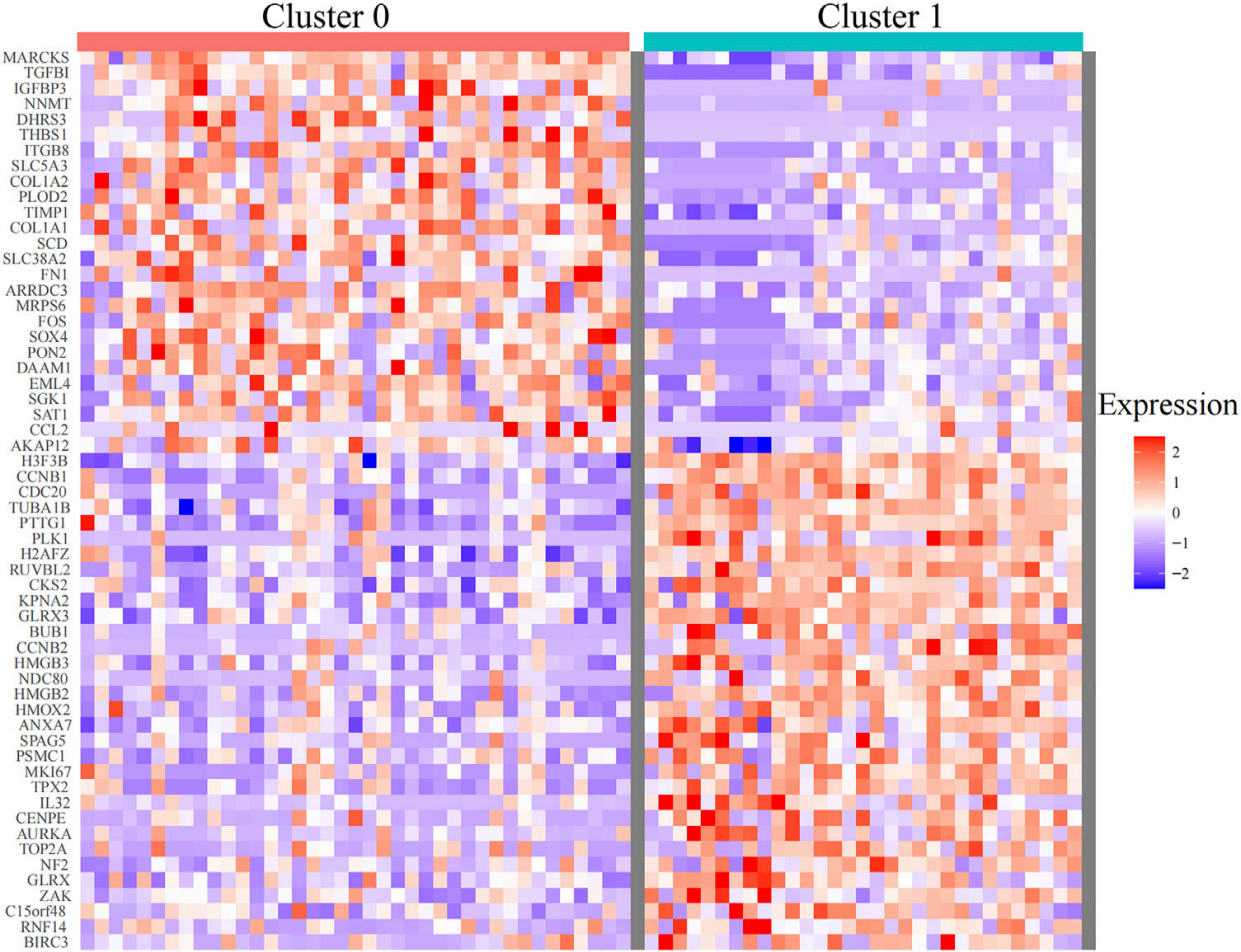
Heatmaps of differentially expressed genes between cluster 0 and cluster 1 identified by TSNE analysis (with adjusted *p* < 0.05). TSNE, t-Stochastic Neighbor Embedding.

Single-cell sequencing datasets of 10 CTCs, 23 LM cells, and 37 PT cells derived from metastatic PDAC patient–derived xenograft (PDX) mouse models were obtained from GSE114704. Quality control of read counts was implemented by “Seurat” (Figures 5A–C). As the expression of the mitochondrial gene is generally low, cells with nFeature > 50 were selected for subsequent analysis. Thereafter, a scatter diagram was plotted to find genes with larger expression alteration in different cells (Figure 5D), and the top 5,000 genes were selected for future analysis. Next, PCA was conducted and 20 principal components (PCs) were obtained. As revealed in the cell distribution in the top two PCs (PC1 and PC2) in Figure 5E, all cell populations could be arranged into two distinct clusters. One of the clusters largely comprised PT cells (in the blue circle), while the other mainly comprised CTCs and LM cells (in the red circle). In addition, cluster analysis was performed on the cells in these 20 PCs, and eventually the first three PCs with the most significant difference were identified (Figure 5F). These three PCs were adopted for TSNE analysis. Similarly, all 70 samples were grouped into two distinct clusters (clusters 0, 1) (Figure 5G). While cluster 0 mainly comprised PT cells (35/39), cluster 1 primarily contained CTCs and LM cells (28/30). The genetic information carried by CTC from metastatic patients is highly similar to the information of metastatic tumor cells, which was consistent with the abovementioned dimensional reduction results (CTCs and metastatic tumor cells clustered together) (Chemi et al., 2019). Therefore, genes between cluster 0 and cluster 1 were differentially analyzed to find marker genes specifically expressed by CTC of metastatic PDAC (Figure 6). Finally, 87 marker genes were identified (|log2FC|>0.5, adjPval<0.05) (Supplementary table S1).

### Functional Enrichment Analysis and Identification of the Hub Gene

**FIGURE 7:**
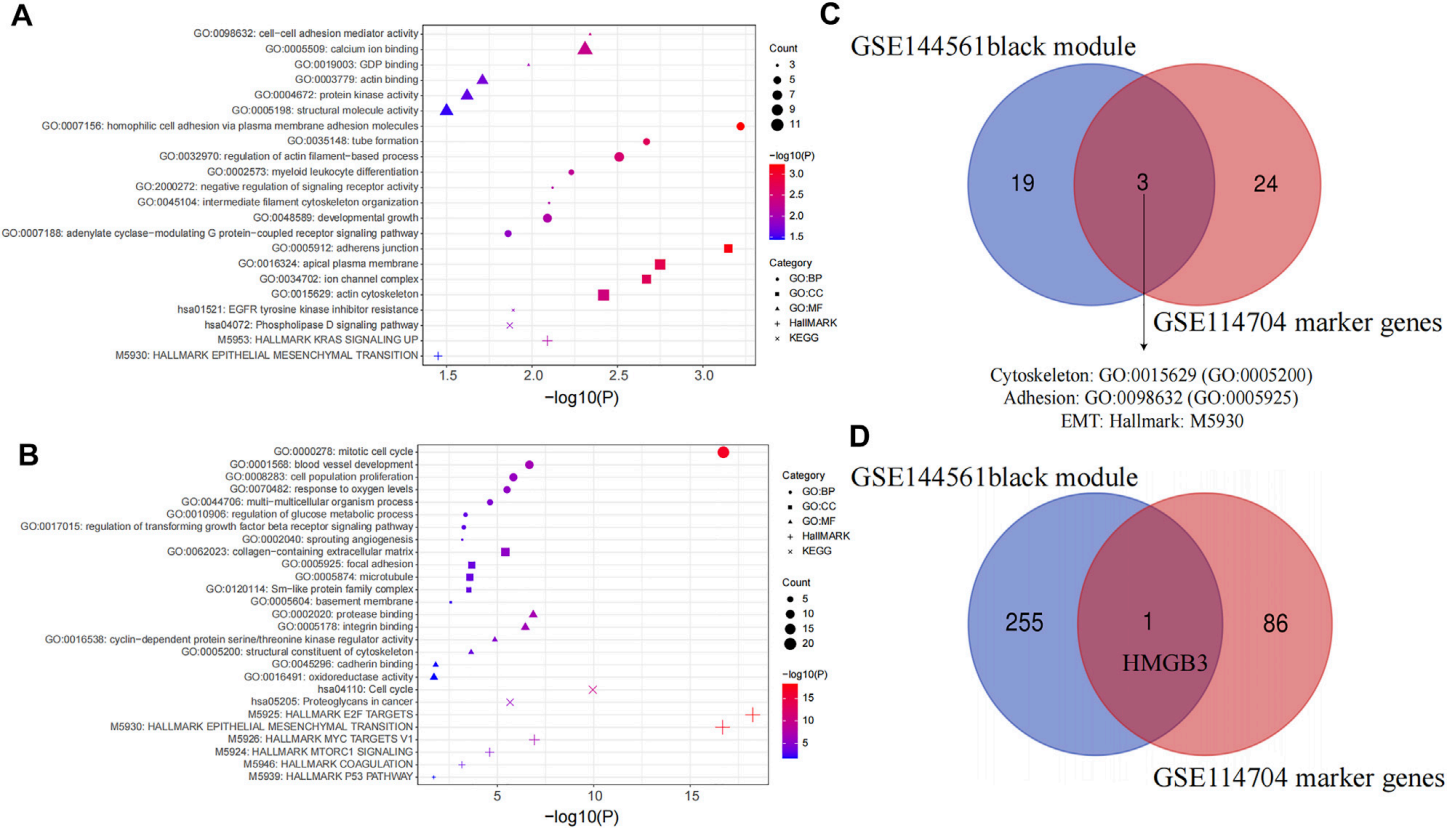
Functional enrichment analysis. **(A)** Functional enrichment analysis of the black module in WGCNA, and **(B)** marker gene between cluster 0 and cluster 1. GO, Gene Ontology; BP, biological processes; CC, cell components; MF, molecular functions; KEGG, Kyoto Encyclopedia of Genes and Genomes. **(C)** Venn plot of enriched pathways in GSE144561 black module and GSE114704 marker genes. **(D)** Venn plot of GSE144561 black module genes and GSE114704 marker genes.

The functional enrichment analysis of hub genes and marker genes was performed by Metascape. BP, CC, MF, Hallmark Gene Sets, and KEGG pathway enrichment analyses were performed separately. Black module genes were functionally enriched in epithelial–mesenchymal transition (Hallmark: M5930, *p* = 0.035), EGFR tyrosine kinase inhibitor resistance (KEGG: hsa01521, *p* = 0.013), and phospholipase D signaling pathway (KEGG: hsa04072, *p* = 0.013), which are crucial pathways for metastasis of PDAC. In addition, cell adhesion (GO:0098632; GO:0007156; GO:0005912; *p* < 0.05), protein kinase activity (GO:0004672, *p* = 0.024), and cytoskeleton (GO:0015629, *p* = 0.004; GO:0045104, *p* = 0.008; GO:0032970, *p* = 0.003) were all significantly enriched again (Figure 7A) While 87 marker genes were functionally enriched in epithelial–mesenchymal transition (Hallmark: M5930, *p* = 0.035), the P53 pathway (Hallmark: M5939, *p* = 0.020) and proteoglycans in cancer (KEGG:hsa05205, *p* < 0.001) are indispensable signals for metastasis of PDAC. In addition, focal adhesion (GO: 0005925; *p* < 0.001), structural constituent of the cytoskeleton (GO:0005200, *p* < 0.001), blood vessel development (GO:0001568; *p* < 0.001; GO:0002040; *p* < 0.001), regulation of the glucose metabolic process (GO:0010906, *p* < 0.001), and regulation of the transforming growth factor beta receptor signaling pathway (GO:0017015, *p* < 0.001) were all significantly enriched again (Figure 7B). Interestingly, the adhesion, cytoskeleton, and EMT pathway were enriched dramatically in both gene sets (GO:0098632; GO:0005925; GO:0015629; GO:0005200; Hallmark: M5930) (Figure 7C). This suggested that CTCs of metastatic PDAC patients might be more likely to undergo EMT or aggregate into CTC clusters. To identify hub genes correlated with metastasis, we screened the coexpressed genes of module genes and marker genes by the Wayne diagram tool, and HMGB3 was identified (Figure 7D).

### Expression and Prognosis of HMGB3

**FIGURE 8:**
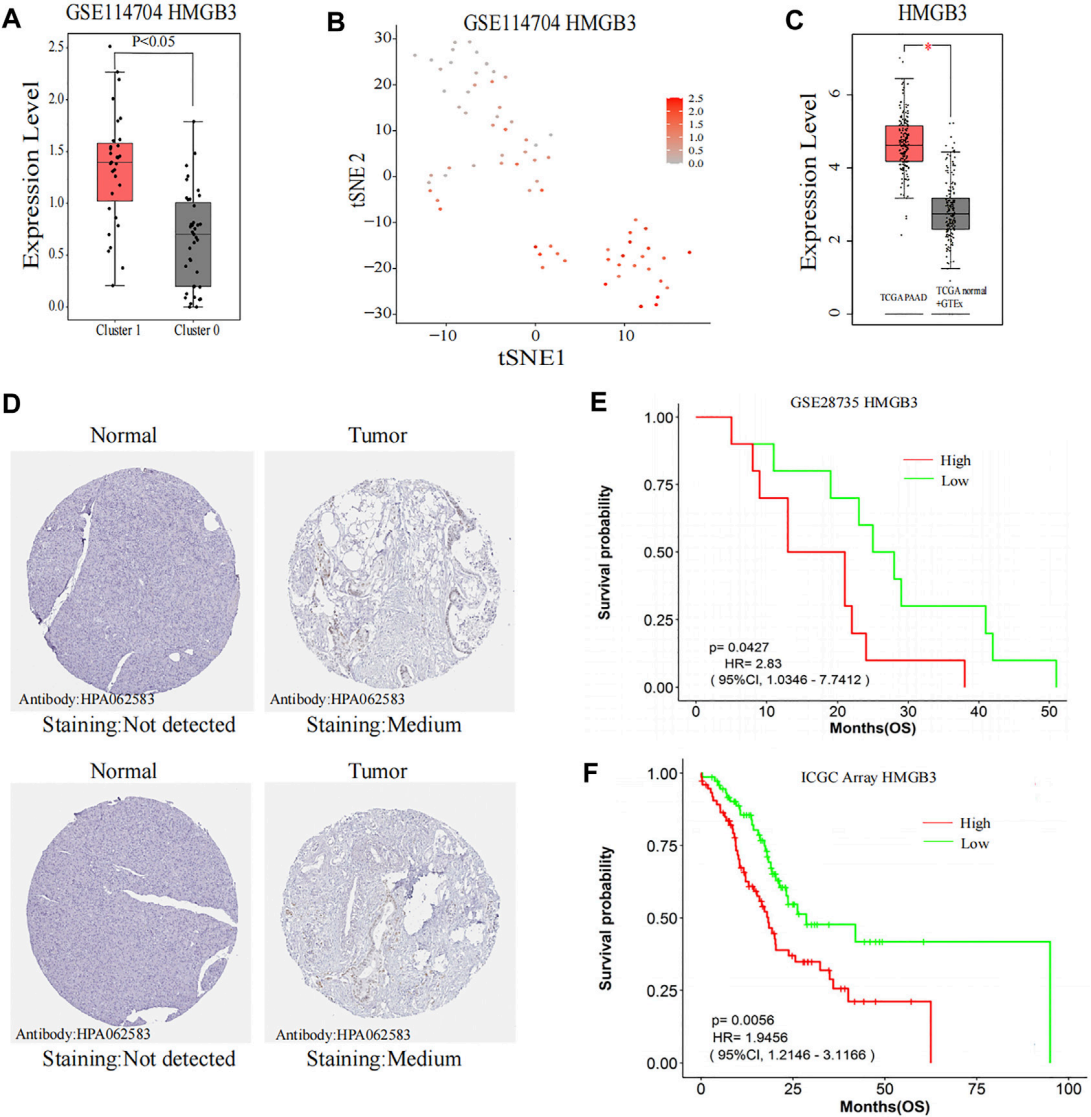
Further research on the hub gene. **(A,B)** Comparison of HMGB3 expression between Cluster 0 and Cluster 1. **(C)** HMGB3 expression in the TCGA pancreatic tumors with corresponding matched normal tissue and GTEx pancreatic tissue. **(D)** Representative immunohistochemistry images of HMGB3 in PAAD and noncancerous pancreatic tissues derived from the HPA database. **(E)** Comparisons of OS curves between high and low expression of HMGB3 in GSE28735. **(F)** Comparisons of OS curves between high and low expression of HMGB3 in ICGC Array. TCGA, The Cancer Genome Atlas; ICGC, International Cancer Genome Consortium; GTEx, The Genotype-Tissue Expression; HPA, the Human Protein Atlas; HMGB3, high-mobility group box 3; PAAD, pancreatic adenocarcinoma; OS, overall survival; HR, hazard ratio; CI, confidence interval.

HMGB3 from the black module has a significant positive correlation with metastasis (*p* < 0.001), and HMGB3 was significantly overexpressed in cluster 1 (CTCs and metastatic tumor cells) as compared to cluster 0 (PT cells) (*p* < 0.001) (Figures 8A,B). Furthermore, through the GEPIA 2 database, HMGB3 was also found to be highly expressed in pancreatic carcinoma compared with the matched normal tissue and the normal pancreatic data from the Genotype-Tissue Expression (GTEx) portal (Figure 8C). Moreover, protein expression levels were similarly compared using the HPA database, and the low protein expression levels of HMGB3 were revealed in normal pancreatic tissues, while medium protein expression levels of HMGB3 were revealed in pancreatic cancer tissues (Figure 8D). We further investigated whether the expression level of HMGB3 correlated with OS in the PAAD by LOGpc. We found that patients with higher HMGB3 expression in GSE28735 and ICGC Array have significantly worse OS (HR = 2.83, *p* = 0.0427; HR = 1.95, *p* = 0.0056) (Figures 8E,F).

### Correlation Analysis of HMGB3 With EMT, CTC Clusters, and Immune Cells

**FIGURE 9:**
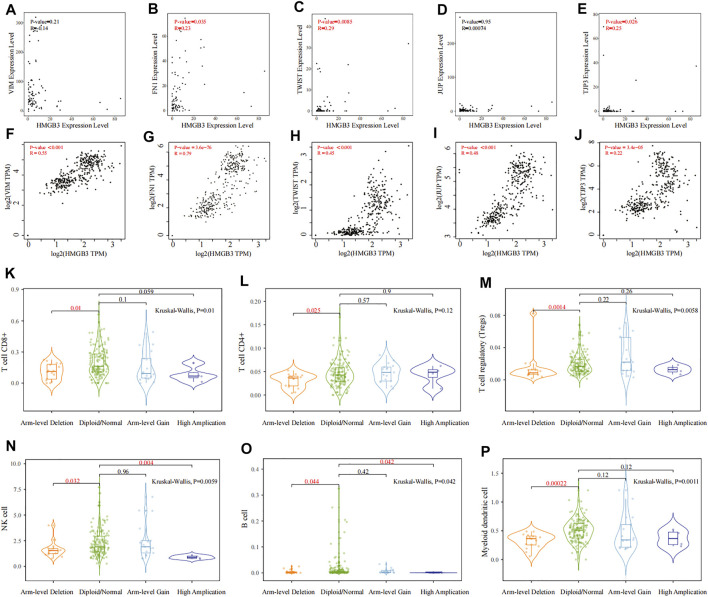
Correlation analysis of HMGB3 with EMT, CTC clusters, and immune cells. **(A–E)** Correlation analysis between HMGB3 expression and VIM, FN1, TWIST, JUP, and TJP3 in GSE14456, respectively. **(F–J)** Relationship between HMGB3 expression and VIM, TWIST, JUP, and TJP3 through the GEPIA 2 database, respectively. **(K–P)** Relationship of HMGB3 mutation with tumor-associated CD8^+^ T cell, CD4^+^ T cell, T cell regulatory, NK cell, B cell, and myeloid dendritic cell infiltrating levels by TIMER2.0, respectively.

Ultimately, we evaluated the correlation between HMGB3 and EMT-related molecules (vimentin, FN1 and TWIST) (Wei et al., 2019; Lin et al., 2021), CTC cluster–related markers (JUP; TJP3) (Harris and Tepass, 2010; Aceto et al., 2014; Gkountela et al., 2019) and immune cells (T cell CD8^+^ T cells, regulatory T cells, NK cell, and dendritic cell) to further confirm the key role of HMGB3 in CTC survival and metastasis. Surprisingly, the expression of HMGB3 was significantly positively correlated with the expression of FN1 (Cor = 0.23, *p* = 0.035), TWIST (Cor = 0.29, *p* = 0.0085), and JUP3 (Cor = 0.25, *p* = 0.026) (Figures 9B,C,E, respectively), but there was no statistical correlation among HMGB3, vimentin, and JUP in GSE14456 (All *p* value > 0.05) (Figures 9A,D, respectively). In addition, through the GEPIA 2 database, it was found that HMGB3 was also significantly positively correlated with vimentin (Cor = 0.55, *p* < 0.001), FN1 (Cor = 0.79, *p* < 0.001), TWIST (Cor = 0.45, *p* < 0.001), JUP (Cor = 0.48, *p* < 0.001), and TJP3 (Cor = 0.22, *p* < 0.001) (Figures 9F–J). Using TIMER2.0, we also attempted to investigate the relationship between HMGB3 mutation and immune cell infiltration. Notably, HMGB3 showed significant relationships with tumor-associated CD8^+^ T cell, CD4^+^ T cell, T cell regulatory, NK cell, B cell, and dendritic cell infiltrating levels, implying that HMGB3 was favorably related to tumor-associated immune cell infiltration in the CTC microenvironment (All *p* value < 0.05) (Figures 9K–P).

## Discussion

In PDAC, metastasis tends to occur early. Metastasis is the major cause of cancer-related death, and the mechanism of metastasis remains unclear. In recent years, CTCs detected and isolated from blood samples of cancer patients have attracted great attention. CTCs are considered to be responsible for tumor metastasis, released from the primary tumor and spread in the peripheral blood circulation between immune cells and red blood cells (Jeong et al., 2018). With the development of CTC detection and RNA-sequencing technology, it is gradually recognized that there is great heterogeneity among CTCs, and not all CTCs could survive in blood and consequently lead to distant metastasis. Previous studies demonstrated that specific phenotypes of CTCs, for example, CTCs with EMT and CTCs expressing tumor stemness genes and CTC clusters possessed stronger ability to survive in blood circulation (Barriere et al., 2014; Fabisiewicz and Grzybowska, 2017; Agnoletto et al., 2019). Those CTCs might be more relevant to metastasis and might have their own unique microenvironment (Fabisiewicz and Grzybowska, 2017). In addition, studies have reported that the genetic mutation information carried by CTCs from metastatic patients is 91% consistent with the information of metastatic tumor cells; hence, analysis based on CTCs may reveal the key targets that hinder tumor metastasis (Chemi et al., 2019). Furthermore, clinically, CTC is easy to be obtained and collected multiple times and could be used to evaluate the effect of chemotherapy for drug resistance screening in real time, which avoids the limitation of traditional pathological examination of solid tumor lesions. Therefore, it is critical for this study to explore the metastasis mechanism of PDAC based on CTCs.

For the comprehensiveness of our study, we selected GSE144561 and GSE114704, where CTC samples are all separated by negative selection (CTC-iChip) to preserve the diversity of CTC phenotypes. Although the Food and Drug Administration (FDA) approved the use of CellSearch (positive selection) in CTC enrichment, it could only enrich CTCs with epithelial cell adhesion molecules (EpCAM) (Bankó et al., 2019). Since CTCs exist in peripheral blood and are inactivated by immune cell attack, metastatic PDAC patients might have distinctive microenvironments that support CTC survival and eventually induce metastasis. To investigate the immune landscape of CTCs, we first analyzed the bulk CTC RNA-sequencing dataset GSE144561. The results intimated that the proportion of Tregs and macrophages in metastatic PDAC was significantly higher than that in localized PDAC, while the proportion of DCs was significantly lower (All *p* < 0.05). Tregs are a subgroup of T cells. As important immunosuppressive cells, Tregs participate in a variety of immune regulatory mechanisms to inhibit immune surveillance, promoting the occurrence, development, and metastasis of pancreatic cancer (Liu et al., 2016). Macrophages could also suppress tumor-associated immunogenicity, promote immune tolerance, and lead to poor prognosis (Zhong et al., 2018). Dendritic cells (DCs), as the antigen-presenting cells (APCs), could recognize, process, and present tumor antigens to T cells, promoting immune antitumor response (Yamamoto et al., 2012). Hence, the higher proportion of Treg and macrophage infiltration and lower proportion of DC infiltration in CTCs might be responsible for protecting CTCs from immune system attack in circulating blood and ultimately leading to metastasis.

Next, we further explored differences in RNA transcriptome expression between localized PDAC and metastatic PDAC. First, we identified DEGs between PDAC patients and healthy donors. This could lower the potential risk that the enrichment of CTCs might be mixed up with nontumor cells (normal cells in the blood), which affects the accuracy of downstream study. Subsequently, DEGs were analyzed in WGCNA analysis based on localized PDAC and metastatic PDAC. We found that the black modules including 256 genes were significantly positively correlated with metastasis and the correlation coefficient was the highest (Cor = 0.58, *p* < 0.001). In addition, PCA and TSNE analysis were performed on scRNA-seq datasets GSE114704, and it was found that CTCs and metastasis cells could be clustered into cluster 1, which was consistent with the statement that CTCs and metastatic cells were homologous clones (Chemi et al., 2019). On the contrary, cluster 0 mainly comprised primary tumor cells. In order to explore genes correlated with metastasis, which are expressed by CTCs, the genes between cluster 0 and cluster 1 were then differentially analyzed and 87 marker genes were obtained.

Functional enrichment analyses were performed on black module genes and marker genes. Interestingly, the adhesion, cytoskeleton, and EMT pathways were enriched dramatically in both gene sets (GO:0098632; GO:0005925; GO:0015629; GO:0005200; Hallmark: M5930). This suggested that CTCs with metastatic characteristics might be more likely to be CTCs with EMT and CTC clusters. Formation of CTC clusters could protect tumor cells from fluid shear stress, anoikis (apoptotic cell death), and immune surveillance; hence, it had 23–50 times increased metastatic ability compared to that of single CTC (Aceto et al., 2014). The previous immune cell infiltration analysis also revealed that the CTC of patients with metastasis had a higher proportion of macrophages, which also suggested that metastatic patients formed more CTC clusters comprising CTC and macrophages. In terms of EMT, it has been considered an important factor leading to metastasis, promoting migration, invasive potential, and resistance to anoikis (Fabisiewicz and Grzybowska, 2017). Then, we intend to identify the key genes for CTC survival and ultimately metastasis. We found that HMGB3 was the only coexpressed gene of hub module genes and marker genes. Interestingly, we found that HMGB3 was, indeed, associated with immune cell infiltration ( CD8^+^ T cells, regulatory T cells, NK cell, and dendritic cell), EMT-related molecules (vimentin, FN1, and TWIST), and CTC cluster–related markers (JUP and TJP3) (All *p* < 0.05). In addition, the RNA and protein expression of HMGB3 in PDAC were significantly higher than those in normal tissue (*p* < 0.05), and the OS of PDAC patients with higher HMGB3 expression was significantly worse in multiple datasets (GSE28735: HR = 2.83, *p* = 0.0427; ICGC Array: HR = 1.95, *p* = 0.0056).

High-mobility group box 3 (HMGB3) belongs to the high mobility group protein B (HMGB) family and can regulate the mechanisms of DNA replication, transcription, recombination, and repair. It also acts as cytokines to mediate responses to infection, injury, and inflammation (Yanai et al., 2011; Lv et al., 2019). HMGB3 has low or no expression in normal adult tissues but high expression in various kinds of tumor tissues, including esophageal cancer, lung cancer, breast cancer, gastric adenocarcinoma, bladder cancer, prostate cancer, and glioma (Gong et al., 2013; Li et al., 2015; Yamada et al., 2018; Gu et al., 2019; Lv et al., 2019; Xie et al., 2020; Yin and Liu, 2020). HMGB3 is considered to be an oncogene and a hub gene in tumor growth, participating in the growth, migration, invasion, immune escape, and even EMT of tumor cells (Fang et al., 2020; Chen et al., 2021). EMT was also found to be advantageous to CTC survival and metastasis in this study. HMGB3 also promotes self-renewal and colony formation of cancer stem cells and cancer cells (Song et al., 2019). In addition, it was shown that HMGB3 protein could bind to chemotherapeutic drug–induced damage DNA, leading to the activation of ATM‐and Rad3‐related protein, thus promoting the drug resistance of tumor cells (Mukherjee et al., 2019; Jiang et al., 2020). Surprisingly, it was consistent that the EGFR tyrosine kinase inhibitor resistance pathway (KEGG: hsa01521, *p* = 0.013) was significantly enriched in the present study. Moreover, the molecular function of HMGB3 is consistent with the conditions (resistance to fluid shear stress, anoikis, and immune surveillance) required for the survival and metastasis of CTC in blood circulation, suggesting that HMGB3 might be a key gene of CTCs in pancreatic cancer aggressiveness.

But, there are few studies on HMGB in pancreatic cancer, and our study is the first to investigate the metastasis mechanism of PDAC based on CTCs. It seems to be promising that the treatment with HMGB3 as an anticancer target could inhibit the survival and metastasis of CTCs and the proliferation of primary tumor cells, which puts forward a new direction for the treatment of PDAC. Our research center has enriched and isolated CTCs by ScreenCell^®^ as previously described (a filtration method that allows the isolation of various CTCs by size, ensuring the diversity of CTCs) (Francescangeli et al., 2021) and is currently conducting research on primary CTC culture. Once we manage to cultivate primary pancreatic CTCs, subsequent cytological experiments would be carried out to further verify the role of HMGB3 in CTCs.

## Conclusion

In conclusion, the results of our study suggest that CTCs of metastatic PDAC have specific immune landscape, RNA expression profile, and functional pathways. HMGB3 is a hub gene associated with the survival and metastasis of CTCs. This study suggests a potentially worthwhile metastasis mechanism of PDAC based on CTCs, further deepening the understanding of PDAC development. Therapeutic agents targeted at HMGB3 in CTCs might provide a novel strategy for the treatment of PDAC.

## Data Availability

The original contributions presented in the study are included in the article/Supplementary Material, further inquiries can be directed to the corresponding author.
