# Supplementary material for: The Crosstalk Between Immune Infiltration, Circulating Tumor Cells, and Metastasis in Pancreatic Cancer: Identification of HMGB3 From a Multiple Omics Analysis
Source: Front Genet. 2022 Jun 8;13:892177. doi: 10.3389/fgene.2022.892177 (PMC9213737; doi:10.3389/fgene.2022.892177)
Supplement: Supplementary file 1 [file Table1.DOCX]

Supplementary Table 1. Marker genes identified by distant clusters in single-cell RNA sequencing dataset GSE114704.

| cluster | gene | pct.1 | pct.2 | avg_log2FC | p_val | p_val_adj |
| --- | --- | --- | --- | --- | --- | --- |
| 0 | H3F3B | 1 | 1 | -1.109343316 | 2.20E-11 | 3.20E-07 |
| 0 | MARCKS | 1 | 0.968 | 2.249524512 | 9.09E-10 | 1.32E-05 |
| 0 | FKBP9 | 1 | 1 | 1.171484264 | 1.52E-09 | 2.22E-05 |
| 0 | CCNB1 | 0.949 | 1 | -1.976607405 | 5.19E-09 | 7.55E-05 |
| 0 | TUBB4B | 0.949 | 1 | -1.242398792 | 7.39E-09 | 0.000107543 |
| 0 | CDC20 | 0.641 | 1 | -1.409992251 | 7.42E-09 | 0.000107953 |
| 0 | MXRA5 | 0.872 | 0.129 | 0.597449694 | 1.04E-08 | 0.00015062 |
| 0 | TGFBI | 1 | 0.774 | 2.197409103 | 1.11E-08 | 0.000160947 |
| 0 | ITGB1 | 1 | 0.935 | 0.999816759 | 1.21E-08 | 0.000175327 |
| 0 | IGFBP3 | 0.949 | 0.29 | 2.114606823 | 1.22E-08 | 0.000177906 |
| 0 | PTPRS | 0.923 | 0.516 | 0.871333351 | 2.48E-08 | 0.000360182 |
| 0 | TUBA1B | 1 | 1 | -0.979219636 | 3.36E-08 | 0.000488731 |
| 0 | COL3A1 | 0.897 | 0.194 | 0.889098277 | 3.63E-08 | 0.000527993 |
| 0 | NNMT | 0.872 | 0.355 | 1.543558803 | 3.66E-08 | 0.000532783 |
| 0 | DEPDC1 | 0.487 | 0.968 | -0.877341782 | 3.76E-08 | 0.000547444 |
| 0 | PTTG1 | 0.974 | 1 | -1.047186178 | 4.70E-08 | 0.000683097 |
| 0 | PLK1 | 0.333 | 0.968 | -1.033144054 | 6.86E-08 | 0.000998473 |
| 0 | LPCAT2 | 0.974 | 0.871 | 0.772095008 | 7.42E-08 | 0.001079718 |
| 0 | AMIGO2 | 0.872 | 0.226 | 0.830747708 | 8.29E-08 | 0.00120541 |
| 0 | H2AFZ | 1 | 1 | -1.121876617 | 9.08E-08 | 0.001320938 |
| 0 | FAP | 0.769 | 0.097 | 0.60986824 | 1.07E-07 | 0.001554779 |
| 0 | DHRS3 | 0.795 | 0.161 | 1.405930704 | 1.14E-07 | 0.001663117 |
| 0 | THBS1 | 0.872 | 0.387 | 1.221114843 | 1.21E-07 | 0.001758223 |
| 0 | TP53 | 0.974 | 0.903 | 0.710082721 | 1.29E-07 | 0.001882762 |
| 0 | RUVBL2 | 0.949 | 1 | -1.081791206 | 1.43E-07 | 0.002078485 |
| 0 | CKS2 | 1 | 1 | -1.034135249 | 1.52E-07 | 0.002216966 |
| 0 | LPP | 0.974 | 1 | 0.962366597 | 1.62E-07 | 0.002363761 |
| 0 | ARHGAP11A | 0.974 | 1 | -0.89233277 | 1.62E-07 | 0.002363761 |
| 0 | FAM3C | 1 | 0.839 | 1.109646854 | 1.96E-07 | 0.002848757 |
| 0 | PPP1CB | 1 | 1 | 1.158214569 | 1.97E-07 | 0.002862753 |
| 0 | KPNA2 | 0.974 | 1 | -1.484106764 | 2.10E-07 | 0.003050654 |
| 0 | FOSB | 0.949 | 0.613 | 0.689763812 | 2.27E-07 | 0.003303198 |
| 0 | PSMA7 | 1 | 1 | -0.697505647 | 2.38E-07 | 0.003462857 |
| 0 | TSC22D1 | 0.974 | 0.613 | 1.550928065 | 2.99E-07 | 0.004349111 |
| 0 | ARL4C | 1 | 0.968 | 1.231585958 | 3.06E-07 | 0.004454631 |
| 0 | AKR1B1 | 0.231 | 0.774 | -0.555805798 | 3.23E-07 | 0.004693449 |
| 0 | TUBB | 1 | 1 | -0.869629486 | 3.26E-07 | 0.004742518 |
| 0 | PSMB5 | 1 | 1 | -0.906288419 | 3.69E-07 | 0.005373128 |
| 0 | MRPL20 | 0.974 | 1 | -0.699664915 | 4.18E-07 | 0.006084294 |
| 0 | GLRX3 | 1 | 1 | -0.980074246 | 4.18E-07 | 0.006084294 |
| 0 | RAN | 1 | 1 | -0.643457293 | 4.45E-07 | 0.006473118 |
| 0 | TUBA1C | 1 | 1 | -0.926806213 | 4.45E-07 | 0.006473118 |
| 0 | LSM3 | 1 | 1 | -0.605431106 | 5.03E-07 | 0.007323927 |
| 0 | ITGB8 | 0.974 | 0.871 | 1.845893555 | 5.67E-07 | 0.008244556 |
| 0 | HN1 | 1 | 1 | -0.860557979 | 6.84E-07 | 0.009949293 |
| 0 | SLC5A3 | 0.974 | 0.677 | 2.339511437 | 6.92E-07 | 0.010065897 |
| 0 | COL1A2 | 0.949 | 0.484 | 2.01331887 | 7.46E-07 | 0.01085802 |
| 0 | PLOD2 | 1 | 0.806 | 1.447112309 | 7.66E-07 | 0.011148763 |
| 0 | TIMP1 | 1 | 0.935 | 1.502910808 | 7.72E-07 | 0.011233218 |
| 0 | SNTB2 | 1 | 0.935 | 0.872549373 | 7.72E-07 | 0.011233218 |
| 0 | SNRPC | 1 | 1 | -0.568620464 | 7.72E-07 | 0.011235711 |
| 0 | AURKB | 0.41 | 0.903 | -0.939661425 | 8.05E-07 | 0.011709428 |
| 0 | NUTF2 | 1 | 1 | -0.662237119 | 8.21E-07 | 0.01193759 |
| 0 | FBN1 | 0.846 | 0.226 | 0.635537689 | 9.46E-07 | 0.013766575 |
| 0 | MMP2 | 0.821 | 0.29 | 0.938765207 | 9.85E-07 | 0.014328097 |
| 0 | TTC3 | 0.974 | 0.935 | 1.091150419 | 1.04E-06 | 0.015178116 |
| 0 | MRPL18 | 0.974 | 1 | -0.878935637 | 1.04E-06 | 0.015191281 |
| 0 | B4GALT1 | 1 | 0.968 | 0.941320641 | 1.18E-06 | 0.017123058 |
| 0 | BUB1 | 0.59 | 0.935 | -1.25406437 | 1.21E-06 | 0.017647405 |
| 0 | MYADM | 1 | 0.935 | 1.073248863 | 1.33E-06 | 0.019285964 |
| 0 | GEMIN6 | 0.769 | 0.968 | -0.780312333 | 1.40E-06 | 0.020361833 |
| 0 | DLAT | 0.872 | 1 | -0.647591246 | 1.40E-06 | 0.020383679 |
| 0 | CCNB2 | 0.615 | 1 | -1.210389271 | 1.41E-06 | 0.020465688 |
| 0 | CLIC1 | 1 | 1 | -0.727375178 | 1.41E-06 | 0.020470196 |
| 0 | COL1A1 | 1 | 0.645 | 3.445225422 | 1.42E-06 | 0.020732132 |
| 0 | SCD | 1 | 0.774 | 1.864850827 | 1.48E-06 | 0.021464581 |
| 0 | TIMM13 | 1 | 1 | -0.566382096 | 1.49E-06 | 0.021719593 |
| 0 | DNMT3A | 0.897 | 0.452 | 0.990007764 | 1.63E-06 | 0.023731747 |
| 0 | SMARCA1 | 1 | 0.903 | 0.946375224 | 1.68E-06 | 0.024421517 |
| 0 | HMGB3 | 0.949 | 1 | -1.037897067 | 1.78E-06 | 0.025917847 |
| 0 | BUB3 | 1 | 1 | -0.994631674 | 1.78E-06 | 0.025923231 |
| 0 | CCNA2 | 0.308 | 0.871 | -0.555786722 | 1.79E-06 | 0.026038831 |
| 0 | PMEPA1 | 0.974 | 0.774 | 0.903717178 | 1.91E-06 | 0.027821192 |
| 0 | CEP55 | 0.667 | 0.968 | -0.90734014 | 2.24E-06 | 0.032576011 |
| 0 | MRPS18C | 1 | 1 | -0.557673041 | 2.25E-06 | 0.032758129 |
| 0 | SLC39A11 | 0.974 | 0.774 | 1.12474447 | 2.28E-06 | 0.033153573 |
| 0 | NDC80 | 0.692 | 0.968 | -1.065391187 | 2.35E-06 | 0.034172634 |
| 0 | CDCA3 | 0.487 | 0.903 | -0.836582504 | 2.37E-06 | 0.034457145 |
| 0 | SLC38A2 | 0.974 | 0.903 | 1.575373912 | 2.45E-06 | 0.035670572 |
| 0 | LOC284454 | 0.974 | 0.935 | 1.27114141 | 2.53E-06 | 0.036764798 |
| 0 | VMP1 | 1 | 1 | 1.037194029 | 2.53E-06 | 0.036794491 |
| 0 | SBDS | 1 | 1 | -0.6532409 | 2.53E-06 | 0.036794491 |
| 0 | LSM5 | 1 | 1 | -0.697275723 | 2.53E-06 | 0.036794491 |
| 0 | STIP1 | 1 | 1 | -0.88645943 | 2.68E-06 | 0.038987633 |
| 0 | CDKN3 | 0.718 | 1 | -0.729989062 | 3.05E-06 | 0.044363435 |
| 0 | PSMD11 | 1 | 1 | -0.825452795 | 3.19E-06 | 0.046345552 |
| 0 | HMGB2 | 0.872 | 1 | -1.098229985 | 3.36E-06 | 0.048888503 |
| 1 | H3F3B | 1 | 1 | 1.109343316 | 2.20E-11 | 3.20E-07 |
| 1 | MARCKS | 0.968 | 1 | -2.249524512 | 9.09E-10 | 1.32E-05 |
| 1 | FKBP9 | 1 | 1 | -1.171484264 | 1.52E-09 | 2.22E-05 |
| 1 | CCNB1 | 1 | 0.949 | 1.976607405 | 5.19E-09 | 7.55E-05 |
| 1 | TUBB4B | 1 | 0.949 | 1.242398792 | 7.39E-09 | 0.000107543 |
| 1 | CDC20 | 1 | 0.641 | 1.409992251 | 7.42E-09 | 0.000107953 |
| 1 | MXRA5 | 0.129 | 0.872 | -0.597449694 | 1.04E-08 | 0.00015062 |
| 1 | TGFBI | 0.774 | 1 | -2.197409103 | 1.11E-08 | 0.000160947 |
| 1 | ITGB1 | 0.935 | 1 | -0.999816759 | 1.21E-08 | 0.000175327 |
| 1 | IGFBP3 | 0.29 | 0.949 | -2.114606823 | 1.22E-08 | 0.000177906 |
| 1 | PTPRS | 0.516 | 0.923 | -0.871333351 | 2.48E-08 | 0.000360182 |
| 1 | TUBA1B | 1 | 1 | 0.979219636 | 3.36E-08 | 0.000488731 |
| 1 | COL3A1 | 0.194 | 0.897 | -0.889098277 | 3.63E-08 | 0.000527993 |
| 1 | NNMT | 0.355 | 0.872 | -1.543558803 | 3.66E-08 | 0.000532783 |
| 1 | DEPDC1 | 0.968 | 0.487 | 0.877341782 | 3.76E-08 | 0.000547444 |
| 1 | PTTG1 | 1 | 0.974 | 1.047186178 | 4.70E-08 | 0.000683097 |
| 1 | PLK1 | 0.968 | 0.333 | 1.033144054 | 6.86E-08 | 0.000998473 |
| 1 | LPCAT2 | 0.871 | 0.974 | -0.772095008 | 7.42E-08 | 0.001079718 |
| 1 | AMIGO2 | 0.226 | 0.872 | -0.830747708 | 8.29E-08 | 0.00120541 |
| 1 | H2AFZ | 1 | 1 | 1.121876617 | 9.08E-08 | 0.001320938 |
| 1 | FAP | 0.097 | 0.769 | -0.60986824 | 1.07E-07 | 0.001554779 |
| 1 | DHRS3 | 0.161 | 0.795 | -1.405930704 | 1.14E-07 | 0.001663117 |
| 1 | THBS1 | 0.387 | 0.872 | -1.221114843 | 1.21E-07 | 0.001758223 |
| 1 | TP53 | 0.903 | 0.974 | -0.710082721 | 1.29E-07 | 0.001882762 |
| 1 | RUVBL2 | 1 | 0.949 | 1.081791206 | 1.43E-07 | 0.002078485 |
| 1 | CKS2 | 1 | 1 | 1.034135249 | 1.52E-07 | 0.002216966 |
| 1 | ARHGAP11A | 1 | 0.974 | 0.89233277 | 1.62E-07 | 0.002363761 |
| 1 | LPP | 1 | 0.974 | -0.962366597 | 1.62E-07 | 0.002363761 |
| 1 | FAM3C | 0.839 | 1 | -1.109646854 | 1.96E-07 | 0.002848757 |
| 1 | PPP1CB | 1 | 1 | -1.158214569 | 1.97E-07 | 0.002862753 |
| 1 | KPNA2 | 1 | 0.974 | 1.484106764 | 2.10E-07 | 0.003050654 |
| 1 | FOSB | 0.613 | 0.949 | -0.689763812 | 2.27E-07 | 0.003303198 |
| 1 | PSMA7 | 1 | 1 | 0.697505647 | 2.38E-07 | 0.003462857 |
| 1 | TSC22D1 | 0.613 | 0.974 | -1.550928065 | 2.99E-07 | 0.004349111 |
| 1 | ARL4C | 0.968 | 1 | -1.231585958 | 3.06E-07 | 0.004454631 |
| 1 | AKR1B1 | 0.774 | 0.231 | 0.555805798 | 3.23E-07 | 0.004693449 |
| 1 | TUBB | 1 | 1 | 0.869629486 | 3.26E-07 | 0.004742518 |
| 1 | PSMB5 | 1 | 1 | 0.906288419 | 3.69E-07 | 0.005373128 |
| 1 | GLRX3 | 1 | 1 | 0.980074246 | 4.18E-07 | 0.006084294 |
| 1 | MRPL20 | 1 | 0.974 | 0.699664915 | 4.18E-07 | 0.006084294 |
| 1 | TUBA1C | 1 | 1 | 0.926806213 | 4.45E-07 | 0.006473118 |
| 1 | RAN | 1 | 1 | 0.643457293 | 4.45E-07 | 0.006473118 |
| 1 | LSM3 | 1 | 1 | 0.605431106 | 5.03E-07 | 0.007323927 |
| 1 | ITGB8 | 0.871 | 0.974 | -1.845893555 | 5.67E-07 | 0.008244556 |
| 1 | HN1 | 1 | 1 | 0.860557979 | 6.84E-07 | 0.009949293 |
| 1 | SLC5A3 | 0.677 | 0.974 | -2.339511437 | 6.92E-07 | 0.010065897 |
| 1 | COL1A2 | 0.484 | 0.949 | -2.01331887 | 7.46E-07 | 0.01085802 |
| 1 | PLOD2 | 0.806 | 1 | -1.447112309 | 7.66E-07 | 0.011148763 |
| 1 | SNTB2 | 0.935 | 1 | -0.872549373 | 7.72E-07 | 0.011233218 |
| 1 | TIMP1 | 0.935 | 1 | -1.502910808 | 7.72E-07 | 0.011233218 |
| 1 | SNRPC | 1 | 1 | 0.568620464 | 7.72E-07 | 0.011235711 |
| 1 | AURKB | 0.903 | 0.41 | 0.939661425 | 8.05E-07 | 0.011709428 |
| 1 | NUTF2 | 1 | 1 | 0.662237119 | 8.21E-07 | 0.01193759 |
| 1 | FBN1 | 0.226 | 0.846 | -0.635537689 | 9.46E-07 | 0.013766575 |
| 1 | MMP2 | 0.29 | 0.821 | -0.938765207 | 9.85E-07 | 0.014328097 |
| 1 | TTC3 | 0.935 | 0.974 | -1.091150419 | 1.04E-06 | 0.015178116 |
| 1 | MRPL18 | 1 | 0.974 | 0.878935637 | 1.04E-06 | 0.015191281 |
| 1 | B4GALT1 | 0.968 | 1 | -0.941320641 | 1.18E-06 | 0.017123058 |
| 1 | BUB1 | 0.935 | 0.59 | 1.25406437 | 1.21E-06 | 0.017647405 |
| 1 | MYADM | 0.935 | 1 | -1.073248863 | 1.33E-06 | 0.019285964 |
| 1 | GEMIN6 | 0.968 | 0.769 | 0.780312333 | 1.40E-06 | 0.020361833 |
| 1 | DLAT | 1 | 0.872 | 0.647591246 | 1.40E-06 | 0.020383679 |
| 1 | CCNB2 | 1 | 0.615 | 1.210389271 | 1.41E-06 | 0.020465688 |
| 1 | CLIC1 | 1 | 1 | 0.727375178 | 1.41E-06 | 0.020470196 |
| 1 | COL1A1 | 0.645 | 1 | -3.445225422 | 1.42E-06 | 0.020732132 |
| 1 | SCD | 0.774 | 1 | -1.864850827 | 1.48E-06 | 0.021464581 |
| 1 | TIMM13 | 1 | 1 | 0.566382096 | 1.49E-06 | 0.021719593 |
| 1 | DNMT3A | 0.452 | 0.897 | -0.990007764 | 1.63E-06 | 0.023731747 |
| 1 | SMARCA1 | 0.903 | 1 | -0.946375224 | 1.68E-06 | 0.024421517 |
| 1 | HMGB3 | 1 | 0.949 | 1.037897067 | 1.78E-06 | 0.025917847 |
| 1 | BUB3 | 1 | 1 | 0.994631674 | 1.78E-06 | 0.025923231 |
| 1 | CCNA2 | 0.871 | 0.308 | 0.555786722 | 1.79E-06 | 0.026038831 |
| 1 | PMEPA1 | 0.774 | 0.974 | -0.903717178 | 1.91E-06 | 0.027821192 |
| 1 | CEP55 | 0.968 | 0.667 | 0.90734014 | 2.24E-06 | 0.032576011 |
| 1 | MRPS18C | 1 | 1 | 0.557673041 | 2.25E-06 | 0.032758129 |
| 1 | SLC39A11 | 0.774 | 0.974 | -1.12474447 | 2.28E-06 | 0.033153573 |
| 1 | NDC80 | 0.968 | 0.692 | 1.065391187 | 2.35E-06 | 0.034172634 |
| 1 | CDCA3 | 0.903 | 0.487 | 0.836582504 | 2.37E-06 | 0.034457145 |
| 1 | SLC38A2 | 0.903 | 0.974 | -1.575373912 | 2.45E-06 | 0.035670572 |
| 1 | LOC284454 | 0.935 | 0.974 | -1.27114141 | 2.53E-06 | 0.036764798 |
| 1 | LSM5 | 1 | 1 | 0.697275723 | 2.53E-06 | 0.036794491 |
| 1 | SBDS | 1 | 1 | 0.6532409 | 2.53E-06 | 0.036794491 |
| 1 | VMP1 | 1 | 1 | -1.037194029 | 2.53E-06 | 0.036794491 |
| 1 | STIP1 | 1 | 1 | 0.88645943 | 2.68E-06 | 0.038987633 |
| 1 | CDKN3 | 1 | 0.718 | 0.729989062 | 3.05E-06 | 0.044363435 |
| 1 | PSMD11 | 1 | 1 | 0.825452795 | 3.19E-06 | 0.046345552 |
| 1 | HMGB2 | 1 | 0.872 | 1.098229985 | 3.36E-06 | 0.048888503 |
