# Supplementary figures and images for: The Crosstalk Between Immune Infiltration, Circulating Tumor Cells, and Metastasis in Pancreatic Cancer: Identification of HMGB3 From a Multiple Omics Analysis
Source: Front Genet. 2022 Jun 8;13:892177. doi: 10.3389/fgene.2022.892177 (PMC9213737; doi:10.3389/fgene.2022.892177)

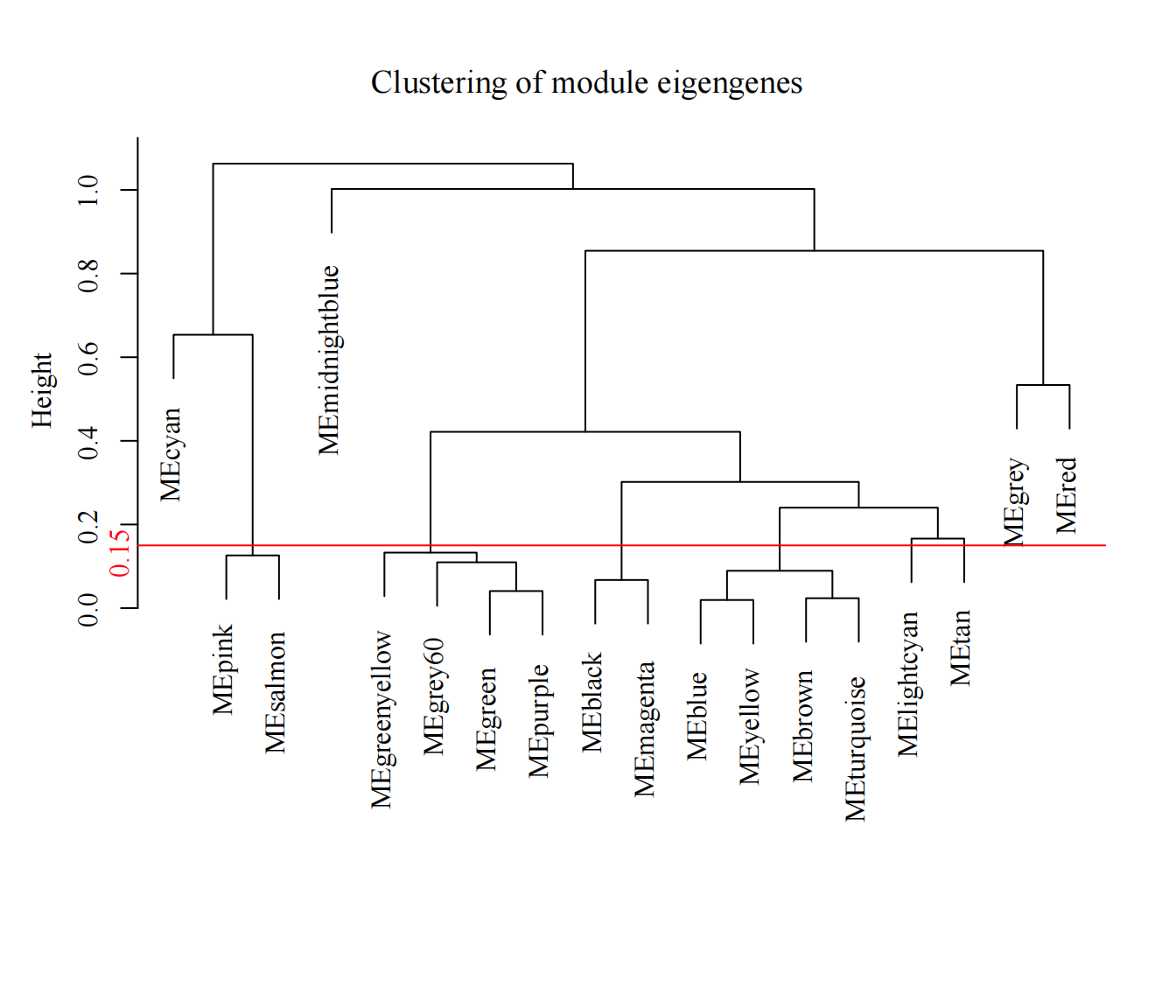

Supplement: Supplementary file 2 [file Image3.TIF]

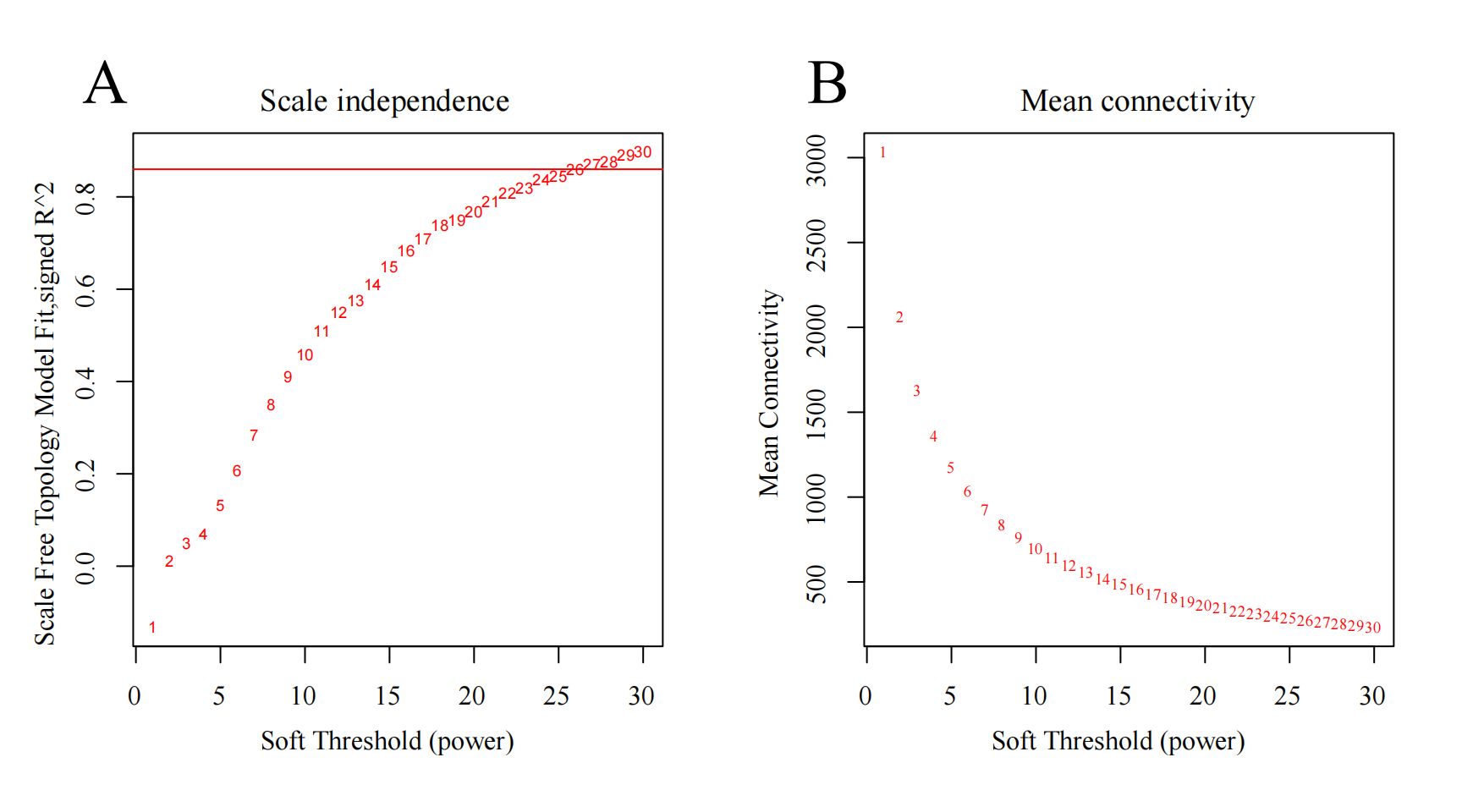

Supplement: Supplementary file 3 [file Image2.TIF]

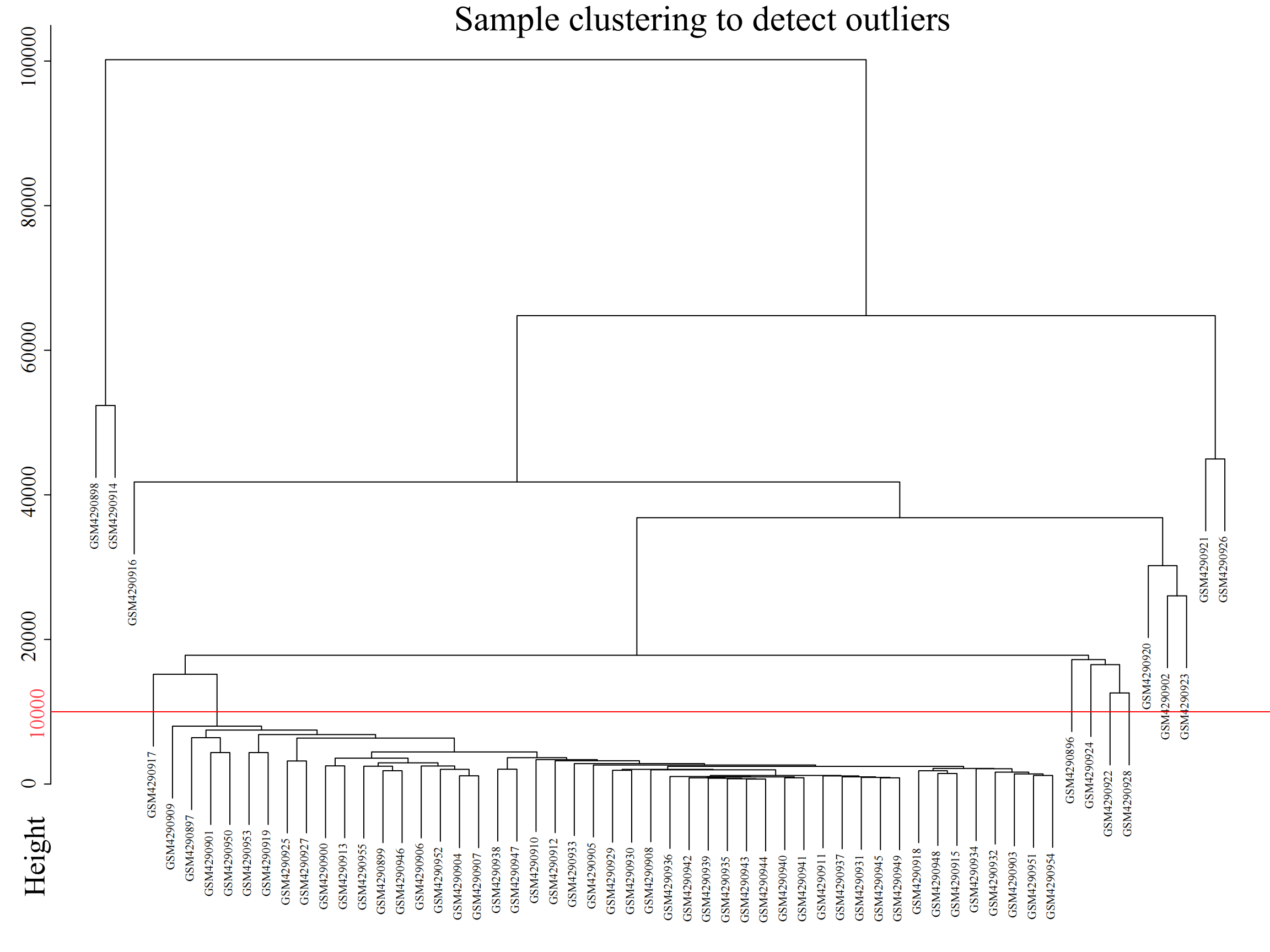

Supplement: Supplementary file 4 [file Image1.TIF]
